# Channeling in helium ion microscopy: Mapping of crystal orientation

**DOI:** 10.3762/bjnano.3.57

**Published:** 2012-07-10

**Authors:** Vasilisa Veligura, Gregor Hlawacek, Raoul van Gastel, Harold J W Zandvliet, Bene Poelsema

**Affiliations:** 1 Physics of Interfaces and Nanomaterials, MESA+ Institute for Nanotechnology, University of Twente, PO Box 217, 7500AE Enschede, The Netherlands

**Keywords:** channeling, crystallography, helium ion microscopy, ion scattering

## Abstract

**Background:** The unique surface sensitivity and the high resolution that can be achieved with helium ion microscopy make it a competitive technique for modern materials characterization. As in other techniques that make use of a charged particle beam, channeling through the crystal structure of the bulk of the material can occur.

**Results:** Here, we demonstrate how this bulk phenomenon affects secondary electron images that predominantly contain surface information. In addition, we will show how it can be used to obtain crystallographic information. We will discuss the origin of channeling contrast in secondary electron images, illustrate this with experiments, and develop a simple geometric model to predict channeling maxima.

**Conclusion:** Channeling plays an important role in helium ion microscopy and has to be taken into account when trying to achieve maximum image quality in backscattered helium images as well as secondary electron images. Secondary electron images can be used to extract crystallographic information from bulk samples as well as from thin surface layers, in a straightforward manner.

## Introduction

The superior resolution of the helium ion microscope (HIM) and its outstanding performance on insulating samples [1–2] make it an interesting tool for materials research. Whilst images based on secondary electrons (SE) can yield an edge resolution down to 0.29 nm [2], backscattered helium (BSHe) images reveal the elemental composition of the specimen. A measurement of the energy of the backscattered helium atoms provides quantitative information on composition [3], and ionoluminescence gives access to electronic properties such as the band structure and the nature of color centers. Unfortunately, to date no experimental procedure has been developed to obtain texture data or crystallographic information systematically in HIM. Especially the latter is an important issue in materials characterization. An important phenomenon that can be exploited in HIM for this purpose is channeling. This well-known process has been studied extensively in the past in the context of ion scattering methods, such as Rutherford backscattering (RBS) and medium- and low-energy ion scattering. Many ion scattering phenomena are well understood for the very high energies of several hundred keV up to MeV that are used in RBS. Although energies in HIM are different and typically range between 5 keV and 40 keV, the existing theories can in fact describe the channeling phenomena with sufficient precision.

Here, we highlight the importance of channeling in the formation of images in HIM. Using gold as an example, we show how the SE yield can be increased by a factor of two. Judicious use of this knowledge allows for an efficient optimization of signal and contrast in HIM images. In the second part of this manuscript we provide an experimental procedure to accurately obtain crystallographic information in HIM. The strong dependence of channeling on the angle of incidence of the beam is used to perform crystal orientation mapping. This procedure provides information that is comparable to electron backscatter diffraction (EBSD). We also show that a fully fledged scattering calculation is not necessary to access this information. Simple geometric considerations are in fact sufficient.

## Experimental

All images were recorded on an ultrahigh vacuum (UHV) Orion Plus helium ion microscope from Carl Zeiss [4]. The microscope is equipped with an Everhardt–Thornley (ET) detector to record SE images. A micro channel plate, which is placed in the beam path below the last lens, is used to record BSHe images. A silicon drift detector measures the energy of backscattered helium atoms and a Gatan MonoCL4 Elite detector measures ionoluminescence. The base pressure of 2 × 10^−9^ mbar allows for extended exposure of the same sample area to the He^+^ ion beam. The near absence of hydrocarbons in the sample chamber effectively reduces carbon build-up in the investigated sample area. High-resolution images were recorded by using the ET detector and a typical primary energy (PE) of 33 keV. To enhance the channeling contrast, some of the data has been recorded with a lower PE of 15 keV. This increases the scattering probability and results in a better signal-to-noise ratio for ion-channeling contrast images. Commercially available polycrystalline gold{111} films on glass, with a chromium interlayer, were flame annealed in a hydrogen flame before the samples were loaded into the vacuum chamber. In addition the samples were cleaned for 15 min in the load lock by using a 10 W air plasma. SRIM-2011 [5] was used to assess the damage caused by the swift helium atoms. The Monte Carlo code was setup to track the full damage cascade and 1 × 10^6^ ions were used. To obtain a measure for the backscattering probability, angle-dependent projections of the crystal lattice were calculated by using a simple geometric model of the crystal slab. The atomic radius of gold was fixed to 0.68 Å and the lattice parameter was 4.078 Å (density 6 × 10^22^ cm^−3^). To speed up the calculations, the thickness of the crystal slab was restricted to 14 layers. This corresponds to a thickness of 3.06 nm and is greater or equal to the information depth in SE images. This in turn depends on the escape depth of SEs in HIM [6]. The crystal slab was tilted and rotated with respect to the (111) surface plane and the 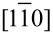 direction, and the blocked area fraction (opacity) of the projection was calculated. To avoid lateral finite size errors, an area of 14 nm^2^ was used for averaging, and border atoms were included with their corresponding area fraction.

## Results and Discussion

### Channeling in helium ion microscopy

**Figure 1:**
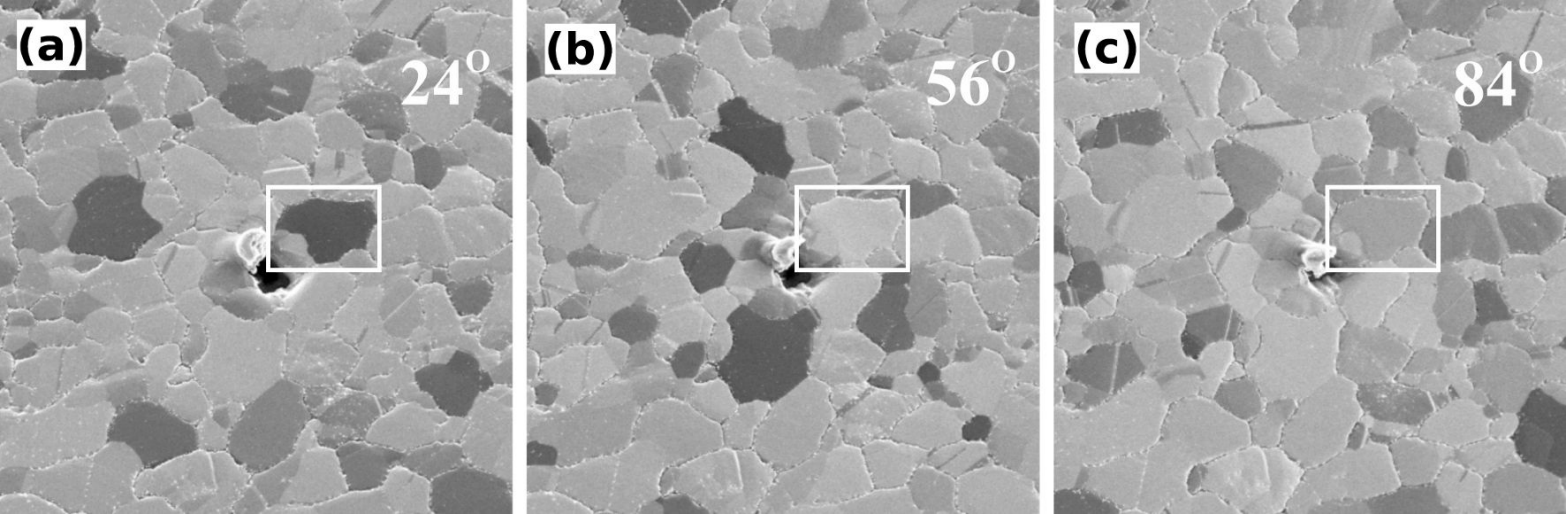
HIM SE images of the hydrogen-flame-annealed polycrystalline Au{111} film taken with a PE of 15 keV and an ion dose of 4.9 × 10^14^ cm^−2^. Relative sample rotation angles around the surface normal are 24°, 56° and 84°. The polar angle is fixed at 35°. FOV: 10 µm.

In Figure 1 SE images of a polycrystalline gold film with a {111} texture are shown. The images with a field of view (FoV) of 10 µm were recorded by using a sample tilt (polar angle) of 35°, a PE of 15 keV and an ion dose of 4.9 × 10^14^ cm^−2^. In Figure 1a individual grains with an average size of 1 µm^2^ can easily be distinguished, not only by their distinct shape, but also through the different gray levels. In Figure 1b and Figure 1c, HIM images are presented that show the same area but for different stage rotation angles (sample rotation is about the [111] surface normal). The gray level of the highlighted grain changes from dark gray to a brighter shade and finally back to a medium shade of gray. The gray level of the other grains changes in an identical sequence, but with different starting points. As we will show below, this allows us to identify the orientation of the individual grains.

**Figure 2:**
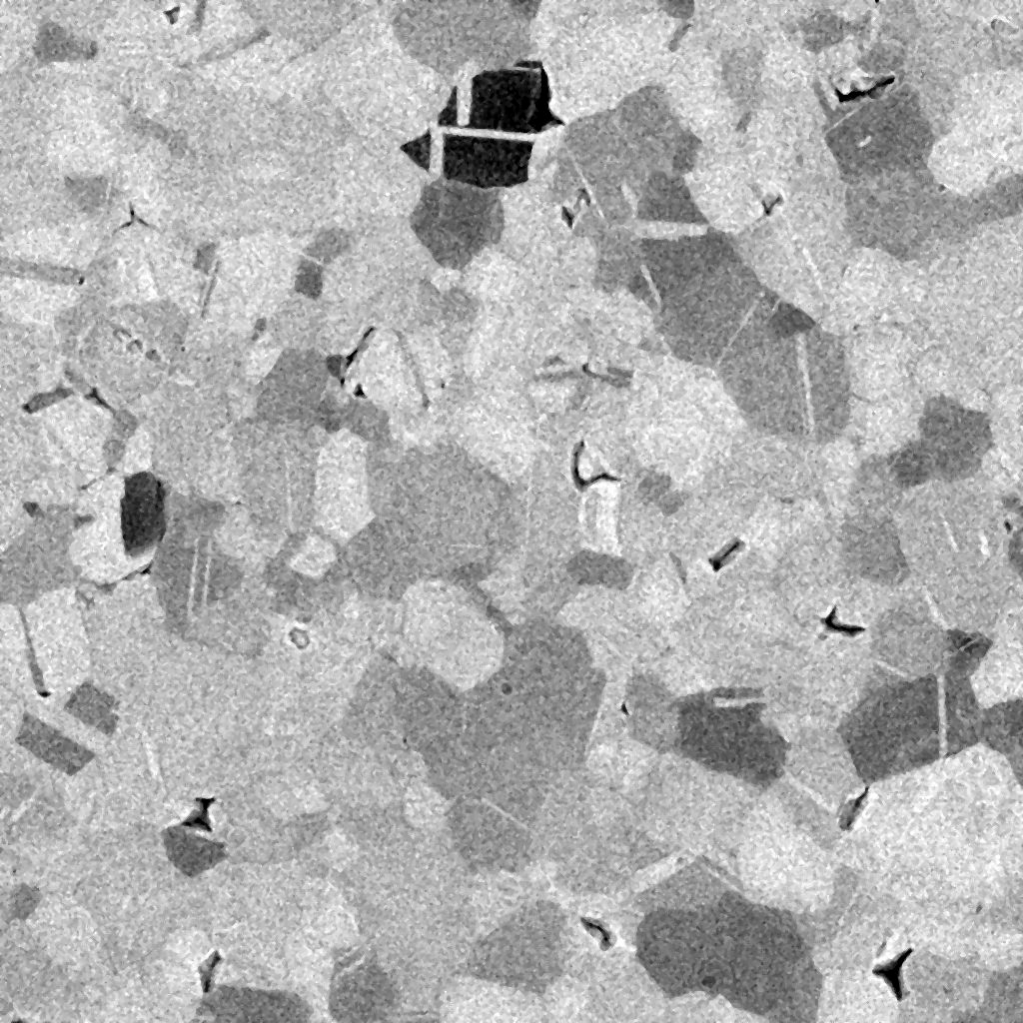
HIM BSHe images of the hydrogen-flame-annealed polycrystalline Au{111} film. A PE of 20 keV and an ion dose of 1.11 × 10^15^ cm^−2^ has been used. The stage tilt was 0°. FOV: 15 µm.

A BSHe channeling contrast image recorded with the MCP detector is presented in Figure 2. Although, there is excellent contrast at the selected acceleration voltage of 20 keV and the mild dose of 1.11 × 10^15^ cm^−2^, the signal-to-noise ratio is considerably worse compared to the SE images presented above. The reason for this is rooted in the low number of ions used per pixel. In the present case only 2375 ions are used per pixel, of which roughly 20% are backscattered, according to SRIM calculations. However, not all of these 500 ions will be counted by the detector. Different to the SE images, BSHe images contain information on the bulk crystallography. The achievable information depth will depend on acceleration voltage and elemental composition of the specimen. However, for gold at 20 keV it is of the order of a few tens of nanometers, and consequently higher than the SE information depth of 2 nm to 3 nm [6].

The stability of the contrast is quite remarkable. With the selected ion dose up to a 100 images can be recorded in the same area. After this, the accumulated dose will have induced an unacceptable number of defects and amorphization will start [7]. In this study, roughly 30 images were recorded, resulting in an ion dose well below the critical threshold. In addition, SE ion-channeling contrast images were used to obtain the data. The information depth in SE images is restricted to the first two or three nanometers below the sample surface. However, ion-induced damage will occur mostly in deeper sample areas and therefore not affect the SE images directly. Using SRIM [5] and a PE of 15 keV into gold, we calculate that 49 vacancies are generated per ion. After a fluence of 4.9 × 10^14^ cm^−2^, necessary to record one image, the defect density at a depth of 3 nm is 4% for the 0 K SRIM simulation. If we assume that about 99% of the defects will heal at a more realistic temperature of 300 K, we obtain a defect density of approximately 1% at a depth of 3 nm after 30 images have been recordeds (fluence: 1.5 × 10^16^ cm^−2^).

**Figure 3:**
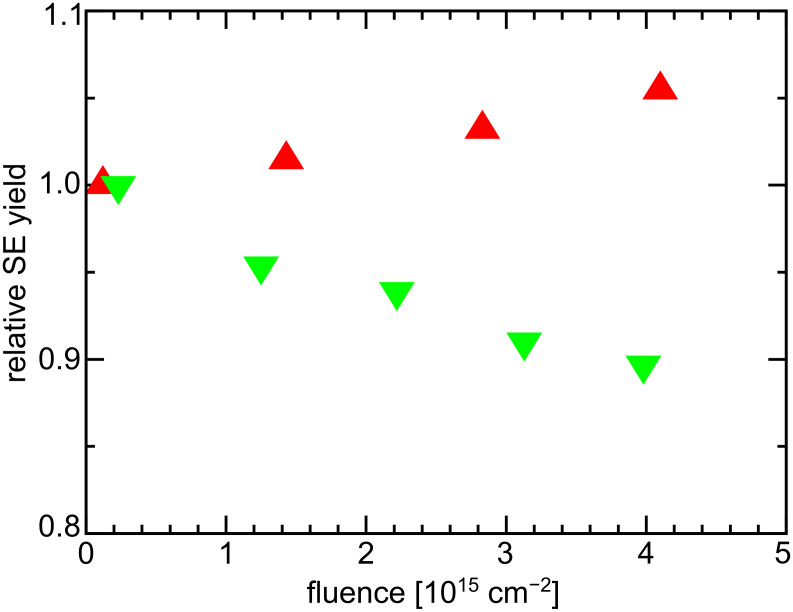
Comparison of contrast evolution in a standard HV and the used UHV HIM. The SE yield, which is proportional to the gray level in SE images, is plotted against the total dose for a HV system (green pointing down triangles) and the used UHV HIM (red pointing up triangles).

The use of an UHV HIM also suppresses the formation of carbon residues on the surface due to hydrocarbon cracking by the energetic helium beam. This is illustrated by a measurement of the SE yields as a function of ion dose presented in Figure 3. A PE of ≈30 keV and an ion dose of 1 × 10^14^ cm^−2^ per image was used to record the data. After a fluence of 4.0 × 10^15^ cm^−2^ the SE yield in a well-maintained standard HV HIM decreased by 10%. However, under identical imaging conditions and for the same total dose, a small 5% increase in SE yield is observed in a UHV HIM. We attribute this slight increase to a mild surface sputtering under the prolonged beam exposure. In general a rougher surface will have a smaller work function, which allows a higher number of SE to escape.

**Figure 4:**
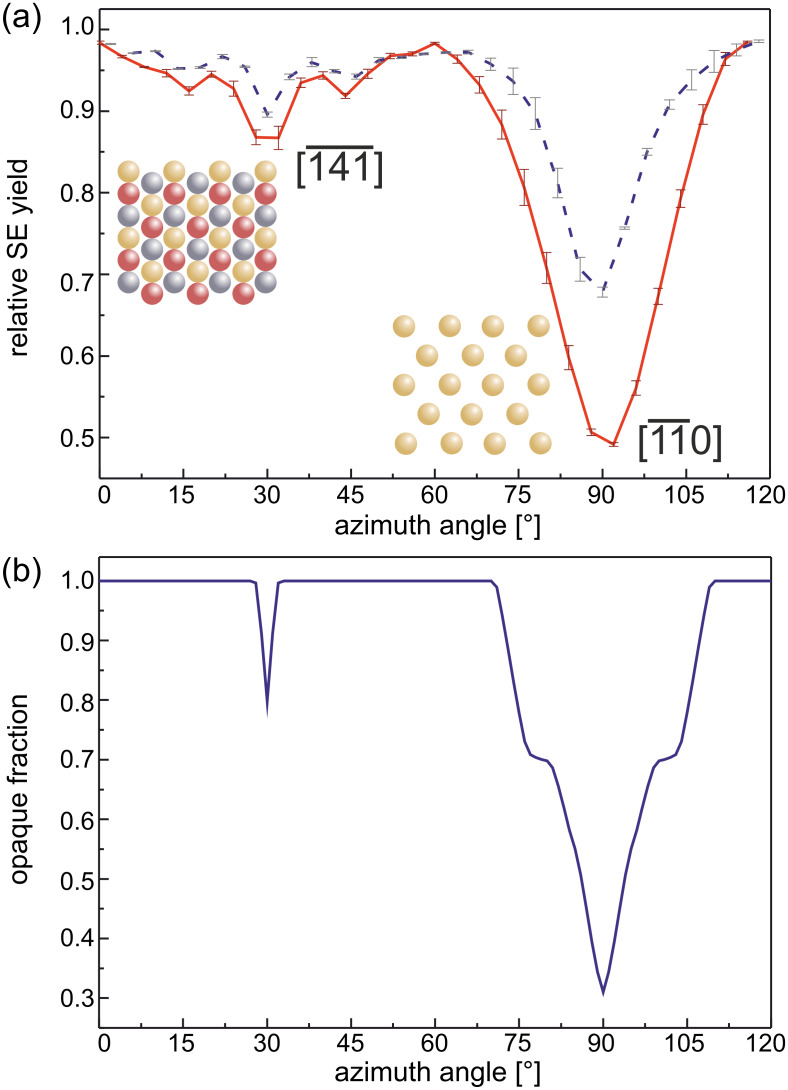
SE yield and opaque fraction for a polar angle of 35° with respect to the (111) plane. The azimuthal angle around the [111] direction is measured with respect to the 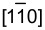 direction. (a) Experimentally obtained dependence of the SE yield on the azimuthal angle. He^+^ PEs are 15 keV (red, solid line) and 33.6 keV (blue, dashed line). The insets present the view of the crystal along the two indicated directions. The color code is as follows: gold: 1st layer, silver: 2nd layer, and red: 3rd layer. (b) Calculated opacity of a gold crystal lattice (14 atomic layers). The used effective atomic diameter is 0.68 Å.

In Figure 4a the average SE yield of several grains is plotted versus the azimuthal angle for two different primary energies. The SE yield of individual grains has been normalized with respect to the maximum intensity and data for the individual grains has been aligned by using the position of the strongest peak. We can understand the angular dependence of the SE yield if we view it as a direct result of the fcc structure of the crystallites in the Au{111} film. The insets in Figure 4a are models of the Au crystal structure. For a given thickness of the sample slab, different fractions of the projected view of the crystal will be occupied by Au atoms. As a result, helium atoms traveling in a direction perpendicular to the projected plane will experience a different channeling probability. If a {111} oriented fcc crystal is tilted by 35° with respect to the incoming beam, for a specific azimuthal orientation, the {110} planes will be parallel to the trajectory of the incoming helium. For symmetry reasons, this configuration can be found every 120°. Particles traveling along the low index <110> directions will undergo a series of small-angle collisions with the atoms of the crystal. This results in a focusing action that allows the particles to travel along the channel [8]. However, to excite electrons in the inner shells of the lattice atoms, hard collisions are necessary. Subsequently, a low number of SE is generated under these conditions [9]. For the images presented in Figure 1 all grains have a {111} plane parallel to the substrate surface. However, the in-plane orientation is random. Consequently, only some grains will be oriented in a channeling direction, while others are not. As a result, a strong grain contrast can be achieved [10–11], in which dark grains are viewed along a channeling direction, while bright crystallites have a blocking orientation. For the marked grain in Figure 1 the stage rotation angles correspond to the azimuthal angle around [111] measured with respect to the 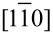 surface direction.

Figure 4b is the result of a simple channeling simulation. A gold fcc crystal slab was tilted by 35° with respect to the (111) plane and rotated around [111] with respect to the 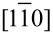 direction. The opacity was then calculated for different azimuthal angles. When compared to the experimental data in Figure 4a it is obvious that the peak positions are reproduced correctly. The shoulders at 75° and 105° are artifacts from the limited crystal slab thickness. In our calculation the fraction of the opaque projected area is directly related to the SE yield measured in the experiment in a qualitative way. In accordance with the experimental results, the calculation predicts minima in the opaque area fraction for the 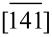 and 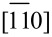 crystal directions.

The depth and width of the minima will depend on the diameter of the simulated atoms. This diameter corresponds to the cross section for scattering and will in turn depend on the actual collision parameters, in particular the primary energy of the incoming particles. The effect of the energy can be seen from Figure 4a: the channeling minima is wider and deeper for the lower energy. The maximum critical angle is determined by the maximum transverse energy [8]. Thus, in the case of lower ion energies the opening angle is bigger, and as a result, more incident ions can be trapped in the channel. In addition, the smaller transverse energy leads to a decrease in the dechanneling probability. Therefore, the lower energies will result in a higher contrast in the SE images.

### Orientation mapping with channeling

The results that we have presented demonstrate that it is possible to obtain crystallographic information directly from SE images in HIM. This information is also accessible from BSHe images; however, the usage of SE has several advantages. First, the required ion dose for a high-quality image with a good signal-to-noise ratio is significantly lower. This is particularly important for light materials. The gold sample that has been used here has a comparatively large backscattering probability for helium atoms. The situation changes, however, for many technologically relevant materials, such as aluminum, iron and silicon. Second, the small information depth of the SEs enables the probing of adlayers and coatings with a thickness in the nanometer range.

**Figure 5:**
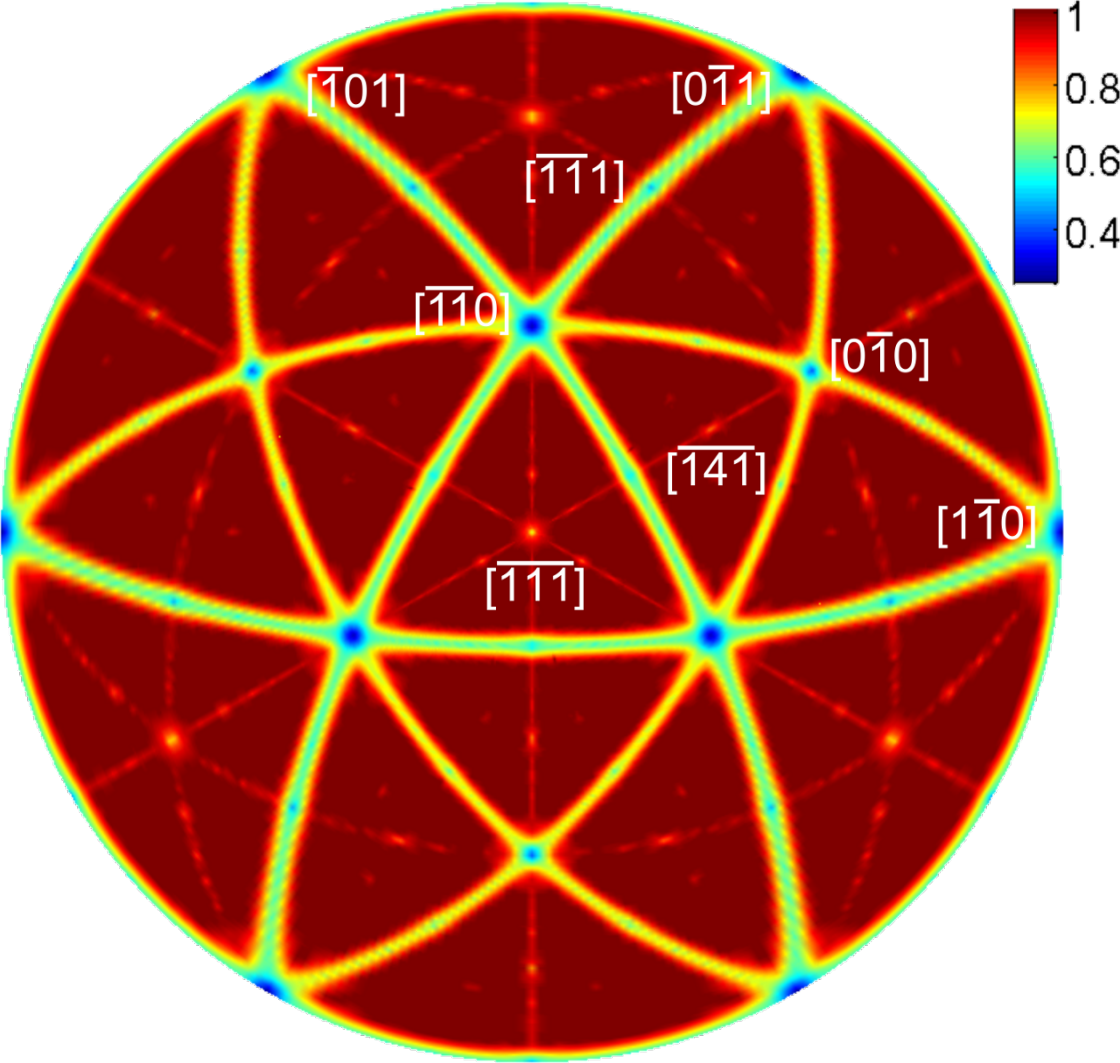
Calculated map of channeling directions for an fcc crystal. The lines connecting the nodes at low-index directions in the polar plot represent channeling directions in the used thin slab of an fcc crystal. The color coding (opaque fraction) allows a qualitative interpretation of the width and depth of the scattering minima.

In Figure 5 we show the calculated positions of the channeling minima of a fcc crystal for imaging with SEs. No exhaustive channeling calculation is necessary to obtain this plot. A simple geometric projection of the first few layers along the beam direction suffices. Despite the fact that the orientation map in Figure 5 resembles a typical stereographic projection of channeling minima [12] or a Laue map [13] for crystal orientations, we briefly highlight the differences. Low-index orientations, such as the <110> directions, are located in the nodes visible in the map. In contrast to a typical stereographic projection of channeling directions, the nodes are connected by a continuous minimum with only small depth undulation. The presence of these shallow lines is directly related to the limited slab thickness used in the calculation. An increase of slab thickness reduces the width and depth of the channeling minima. While stereographic projections give measures for the depth of channeling minima, this information has to be carefully reviewed in the present projection of the opaque crystal fraction map. The width and depth of the minima also depends on the ratio of the nearest-neighbor distance and effective atom radius. In this case we have simply used the ionic radius of gold. However, the parameters that were used create an excellent match between the experimental and simulated data (see Figure 4). In particular, the presence of the zone lines allows for an easy alignment of experimental maps to the calculated data, and the successive identification of crystal orientation.

**Figure 6:**
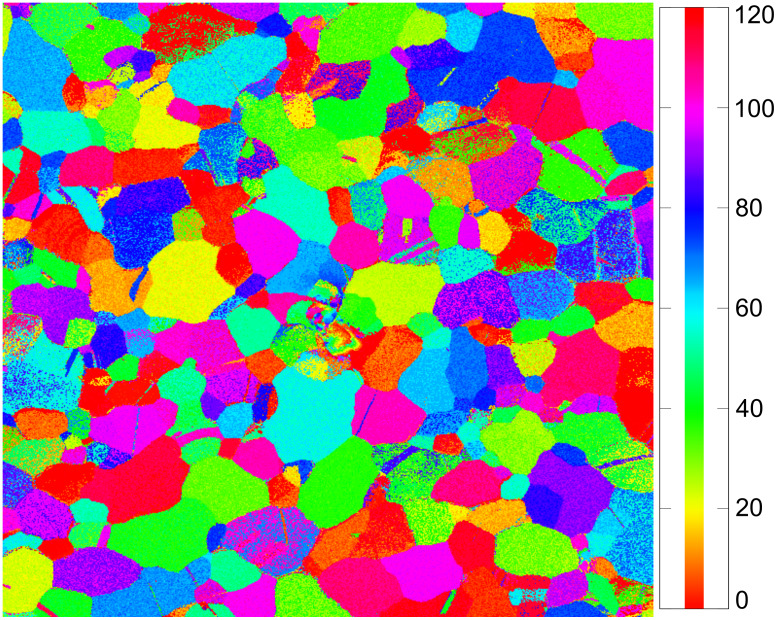
Color-coded orientation map of a polycrystalline gold film. The colors indicate the azimuthal angle around the [111] surface normal for a 35° sample tilt at which a <110> direction is aligned with the incoming helium ion beam. Parts of the data set used are presented in Figure 1.

The map presented in Figure 5 was used to index several grains. A color code representing the different orientations was used to illustrate the different in-plane orientations of the grains. Such a color-coded map is shown in Figure 6. Figure 6 shows the orientation for the grains imaged in Figure 1. The grain that is highlighted in Figure 1 is darkest for a 24° rotation angle. This corresponds to a yellow-green color in Figure 6. The color scale ranges from a 0° to 120° azimuthal rotation around [111]. Consequently, the color of an individual grain corresponds to the azimuthal angle for which a <110> direction in this gold grain is parallel to the beam. For the marked grain the stage rotation angle for this condition is 24°.

## Conclusion

We have demonstrated the importance of channeling in HIM using polycrystalline gold films with a <111> texture. To quantitatively explain the orientation-dependent changes in contrast it is not necessary to perform a full calculation of the scattering process. A straightforward projection of the crystal lattice is sufficient to identify low-index channeling directions. Such a map was calculated for an fcc lattice and used to determine the orientation of all gold crystallites in the FoV. The effect is observed in both types of HIM images. It is, however, particularly useful with SE images. Because of the limited information depth of SE ion-channeling contrast images, crystallographic data from thin adlayers can be obtained. BSHe ion-channeling contrast images, on the other hand, yield similar bulk crystallographic information. This adds a new capability to helium ion microscopy. The possibility to obtain crystallographic information on a per pixel basis strengthens the applicability of HIM for materials characterization.
